# Impact of Statins on Cognitive Deficits in Adult Male Rodents after Traumatic Brain Injury: A Systematic Review

**DOI:** 10.1155/2014/261409

**Published:** 2014-07-23

**Authors:** Weijun Peng, Jingjing Yang, Bo Yang, Lexing Wang, Xin-gui Xiong, Qinghua Liang

**Affiliations:** ^1^Institute of Integrated Medicine, Xiangya Hospital, Central South University, No. 87 Xiangya Road, Changsha, Hunan 410008, China; ^2^Key Laboratory of Chinese Gan of State Administration of Traditional Chinese Medicine, Changsha 410008, China; ^3^Institute of Integrated Medicine, Hunan Cancer Hospital, Changsha 410013, China; ^4^The Affiliated Cancer Hospital of Xiangya School of Medicine, Central South University, Changsha 410013, China

## Abstract

The efficacy of statin treatment on cognitive decline is controversial, and the effect of statins on cognitive deficits in individuals with traumatic brain injury (TBI) has yet to be investigated. Therefore, we systematically reviewed the effect of statins on cognitive deficits in adult male rodents after TBI. After identifying eligible studies by searching four electronic databases on February 28, 2014, we assessed study quality, evaluated the efficacy of statin treatment, and performed stratified metaregression and metaregression to assess the influence of study design on statin efficacy. Eleven studies fulfilled our inclusion criteria from a total of 183 publications. The overall methodological quality of these studies was poor. Meta-analysis showed that statins exert statistically significant positive effects on cognitive performance after TBI. Stratified analysis showed that atorvastatin has the greatest effect on acquisition memory, simvastatin has the greatest effect on retention memory, and statin effects on acquisition memory are higher in closed head injury models. Metaregression analysis further showed that that animal species, study quality, and anesthetic agent impact statin effects on retention memory. We conclude that statins might reduce cognitive deficits after TBI. However, additional well-designed and well-reported animal studies are needed to inform further clinical study.

## 1. Introduction

Traumatic brain injury (TBI) is a leading cause of death and disability in industrialized countries and is the leading cause of long-term disability in children and young adults worldwide [1]. One of the most significant disabilities associated with TBI is short- and long-term cognitive deficits [2]. Approximately 65% of patients with moderate to severe TBI report long-term problems with cognitive functioning, and as many as 15% with mild TBI have persistent problems that often include cognitive deficits [3, 4]. These deficits interfere with work, relationships, leisure, and daily living activities, exacting a personal and economic cost that is difficult to quantify [4]. However, despite substantial efforts, few therapeutic options exist to prevent or alleviate cognitive dysfunction after TBI in humans [5, 6].

Statins (3-hydroxy-3-methylglutaryl-coenzyme A reductase inhibitors) are implicated in stroke, Alzheimer's disease, and multiple sclerosis [7] and constitute potential treatment options for TBI due to their pleiotropicity [8]. In experimental TBI, simvastatin increases neurogenesis and suppresses apoptosis [9, 10], ameliorates secondary brain damage [11], and attenuates microglial and astroglial activation [12]. Both simvastatin and atorvastatin increase neurogenesis and inhibit neuronal death [13], and atorvastatin also reduces brain edema [14]. Lovastatin improves histological outcome and reduces inflammation [15]. Furthermore, simvastatin, lovastatin, and atorvastatin restore cognitive deficits caused by TBI [13, 16]. However, there is no systematic evidence available that statins improve cognition in humans with TBI. Moreover, as with all drugs, statins can exert undesirable effects. In 2012, the U.S. Food and Drug Administration issued a statement on cognitive impairment as a potential adverse effect of statins [17], with myopathy being the most well-characterized adverse sequelae [18]. Also, longitudinal studies (both randomized trials and observational studies) of the effects of statins on cognition in individuals without dementia have yielded negative results [19–21].

Although statins show promise for treating cognitive impairment caused by TBI, further clinical trials are needed. Furthermore, given the controversy regarding the effect of statins on cognition, a robust and systematic summary of existing data may assist in the design of clinical trials. Therefore, we investigated the efficacy of statins in treating cognitive deficits in experimental animal models of TBI and explored the impact of study design and quality on reported outcome.

## 2. Materials and Methods

### 2.1. Search Strategy and Study Selection

We searched four electronic databases (PubMed, Medline, Ovid, and ScienceDirect; February 28, 2014) for studies that examined pharmacological treatments for cognitive, behavioral, and motor problems in rodents after TBI. The key search terms (Table 1) were kept broad to capture all potentially relevant articles. Reference lists from the resulting research articles and reviews were used to identify further relevant publications.

To be included in this meta-analysis, a study had to meet several inclusion criteria (Table 2). Three investigators assessed titles and abstracts and obtained copies of articles that described controlled studies of statins in animal models of TBI to determine their eligibility for inclusion. Disagreements among investigators were resolved by consensus after discussion.

### 2.2. Data Extraction

Two investigators extracted information about the studies including animal species, sample size, type of TBI model, main experimental groups, substances used as experimental and control treatments, method/dose/timing of statin administration, type of anesthetic agent, and time of outcome assessment. Disagreements between investigators were resolved by consensus after discussion.

The Morris water maze (MWM) was used to assess cognition. When cognition was assessed at different times after TBI, only the last day was considered. Cumulative statin dose was taken into consideration when comparing neurobehavioral outcomes among studies.

In cases of missing data, we contacted the authors and requested the additional information. If data were expressed only graphically, numerical values were requested from the authors; if a response was not received, digital ruler software was used to estimate numerical values from the graphs. If required data were not presented or obtainable, the study was excluded from analysis.

### 2.3. Methodological Quality of Studies

The methodological quality of individual studies was assessed based on a checklist modified from the Collaborative Approach to Meta-Analysis and Review of Animal Data from Experimental Studies (CAMARADES) as previously described with minor modification [22, 23]. The checklist was comprised of 10 items: (1) peer reviewed publication; (2) presence of randomization of subjects into treatment groups; (3) assessment of dose-response relationship; (4) blinded assessment of behavioural outcome; (5) monitoring of physiological parameters such as body temperature; (6) calculation of necessary sample size to achieve sufficient power; (7) statement of compliance with animal welfare regulations; (8) avoidance of anaesthetic agents with marked intrinsic neuroprotective properties (e.g., ketamine); (9) statement of potential conflicts of interest; (10) use of a suitable animal model. One point was given for evidence of each quality criterion (Table 3).

### 2.4. Statistical Analysis

In line with the* Cochrane Handbook for Systematic Reviews of Interventions,* the global estimated effect of statin treatment on cognitive outcome was determined by calculating standardized mean difference (SMD; equal to the difference in mean outcome between groups divided by the standard deviation of outcomes among participants, reported in units of standard deviation) and 95% confidence intervals (CI) using a random effects model to avoid heterogeneity [23]. SMD is used as a summary statistic in meta-analyses when studies assess the same outcome but measure the outcome in a variety of ways (e.g., multiple studies measuring depression but using different psychometric scales). Within- and between-study variation or heterogeneity was assessed using Cochran's *Q*-statistic [32, 33], with a significant *Q*-statistic (*P* < 0.10) indicating heterogeneity among studies. Heterogeneity was also assessed using the *I* ^2^ metric, with higher values denoting a greater degree of heterogeneity (0–40%: little heterogeneity; 30–60%: moderate heterogeneity; 50–90%: substantial heterogeneity; 75–100%: considerable heterogeneity). *I* ^2^ values ≤50% indicate acceptable heterogeneity among studies [34]. For studies comparing different doses and/or times of drug administration with a single control group, we compared control group data with pooled data from all experimental groups.

Stratified meta-analysis was used to explore the influence of the type of statin, dose, study quality, animal species, type of TBI model, anesthetic agent, and route of drug delivery on estimated effect size [35].

Differences in mean effect sizes were assessed partitioning heterogeneity using the *χ* ^*2*^ distribution with *n* − 1 degrees of freedom (df). Bonferroni correction was used to adjust significance levels for multiple comparisons (declared significance = 1− (1−denoted significance) ∧ (1/number of comparisons)), yielding critical *P* values of 0.0047 for acquisition memory and 0.0043 for retention memory [36, 37].

Metaregression analyses were conducted to reveal potential sources of heterogeneity, as described in a previous study [38]. Covariates included the type of statin, dose, quality of the study, animal species, type of TBI model, anesthetic agent, and route of drug delivery. Due to limited power of our metaregression analyses, we incorporated each covariate separately into the regression model.

The presence of small effect sizes was investigated using funnel plots and Egger's tests. For Egger's tests, a *P* value of <0.10 was considered to indicate the presence of small effect sizes [32].

All statistical analyses were performed using Review Manager (version 5.2) and Stata software (version 12.0).

## 3. Results

### 3.1. Study Inclusion

**Figure 1:**
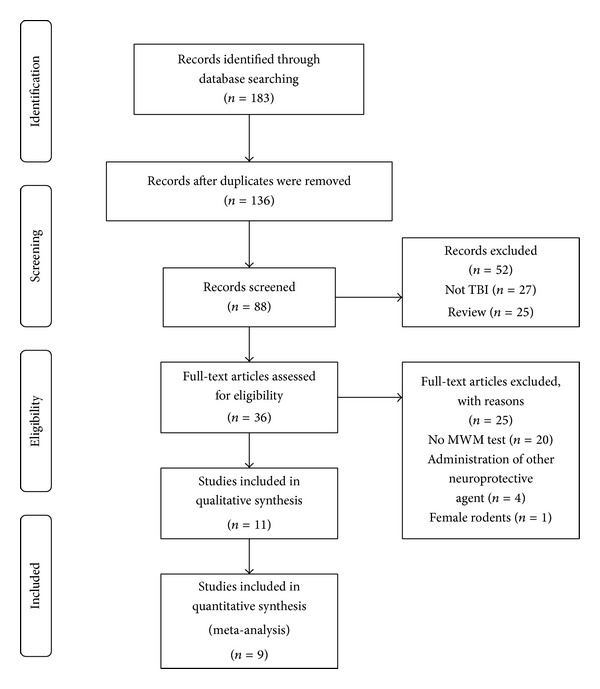
Flow diagram of study search process.

A total of 183 publications were identified, of which 11 met our inclusion criteria [10, 13, 16, 24–31]. Of these, two were excluded from analysis because they did not report sample size [24, 28]. Thus, our meta-analysis is based on nine publications, which include 11 comparisons of acquisition memory and 12 comparisons of retention memory (Figure 1).

### 3.2. Characteristics of Study

Of the 11 included studies (Table 4), three were published in Chinese academic journals [29–31]. Controlled cortical impact injury [10, 13, 16, 24–26] and fluid percussion injury [27, 29–31] were the most frequently used animal models of TBI. Seven studies used rats, three studies used nontransgenic mice, and one study [24] used transgenic mice. Atorvastatin, simvastatin, rosuvastatin, lovastatin, and pravastatin were administered as experimental treatments in doses of 1, 2, 3, or 20 mg/kg/day via oral gavage or subcutaneous injection. All studies used the MWM to assess cognitive function after TBI.

### 3.3. Methodological Quality of Studies

Overall, the median quality score for the 11 included studies was poor (5; interquartile range: 4–7), with scores ranging from 3 to 8. No studies received a score of 0, and four studies [10, 13, 25, 26] received scores indicating high quality (7–10 points). One study [25] did not report randomization of animals into treatment groups. Six studies did not report monitoring of physiological parameters during surgical procedures (although the majority of remaining studies only monitored body or rectal temperature). Only one study [25] assessed dose-response relationships and contained a statement of potential conflict of interests. Four studies [39–42] failed to state that outcome measures were made by experimenters who were blind to animal treatment. Moreover, no study described calculation of necessary sample size.

### 3.4. Overall Efficacy

**Figure 2:**
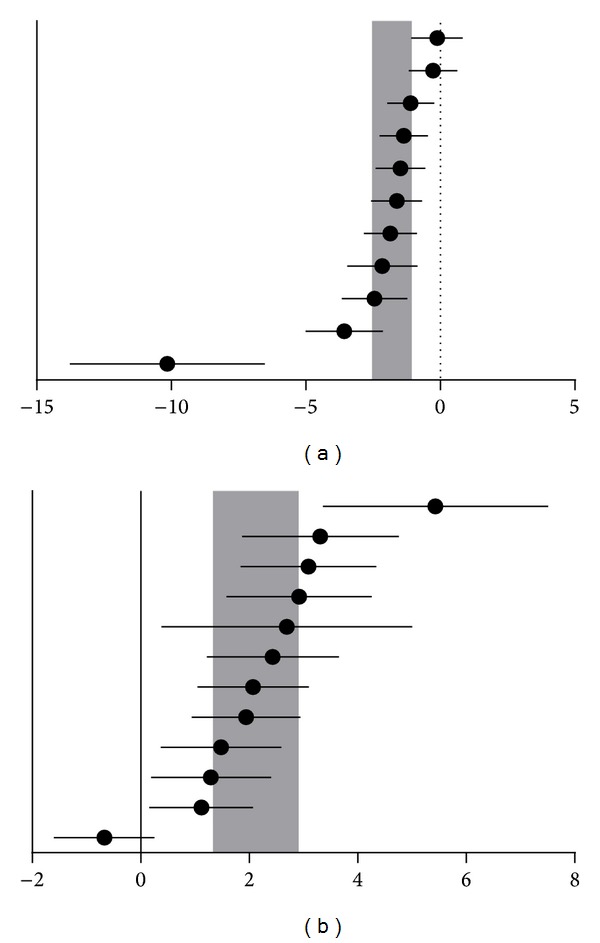
Effects of statins on acquisition memory (a) and retention memory (b). Horizontal lines represent the mean estimated effect size and 95% CI for each comparison. Vertical gray bars represent the 95% CI of the pooled estimated effect size.

For acquisition memory, the global estimated effect of statins was −1.81 (95% CI: −2.54 to 1.07, *P* < 0.0001), with significant heterogeneity among studies (*χ* ^*2 *^= 49.81, df = 10, *P* < 0.0001, *I* ^2^ = 80%; Figure 2(a)). For retention memory, the global estimated effect of statins was 2.12 (95% CI: 1.33 to 2.9, *P* < 0.0001), with significant heterogeneity among studies (*χ* ^*2*^ = 55.33, df = 11, *P* < 0.0001, *I* ^2^ = 80%; Figure 2(b)).

### 3.5. Stratified Meta-Analysis

In a stratified analysis, trials are grouped according to a particular feature or characteristic and separate meta-analyses are conducted for the trials within each subgroup. The overall summaries of each subgroup can then be inspected for evidence of variation in the effects of the intervention, which would suggest that the stratifying characteristic is an important source of heterogeneity and may moderate treatment efficacy [43].

To compare the efficacy of different types of statins, we examined the protective effects of simvastatin, atorvastatin, and rosuvastatin administration on acquisition memory. We did not include pravastatin and lovastatin in this analysis because of limited data. For retention memory, the effects of simvastatin and atorvastatin administration were examined, and rosuvastatin, pravastatin, and lovastatin were excluded because of limited data. Atorvastatin treatment had a greater beneficial effect on acquisition memory (−4.55, 95% CI: −7.33 to −1.36) compared with simvastatin (−1.85, 95% CI: −2.38 to −1.31) or rosuvastatin (−1.28, 95% CI: −1.29 to −0.65) treatment. For retention memory, simvastatin administration (2.87, 95% CI: 1.46 to 4.28) had a greater beneficial effect than rosuvastatin administration, although simvastatin effects showed significant heterogeneity among studies (*I* ^2^ = 77%, *P* < 0.01). No significant differences among types of statins were observed (*P* = 0.08 for acquisition memory and *P* = 0.48 for retention memory, resp.).

**Figure 3:**
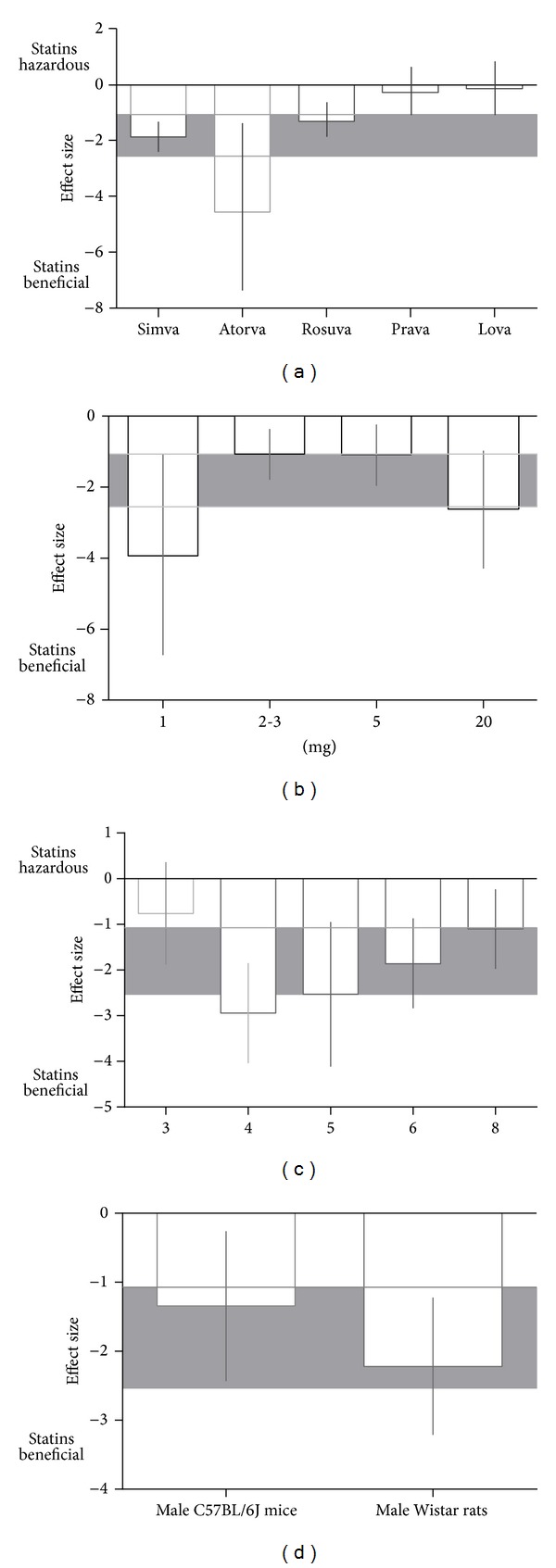
Effect size for acquisition memory stratified by (a) type of statin, (b) dose, (c) quality of study, and (d) animal species. Grey bands represent the 95% CI for the global estimated effect size.

**Figure 4:**
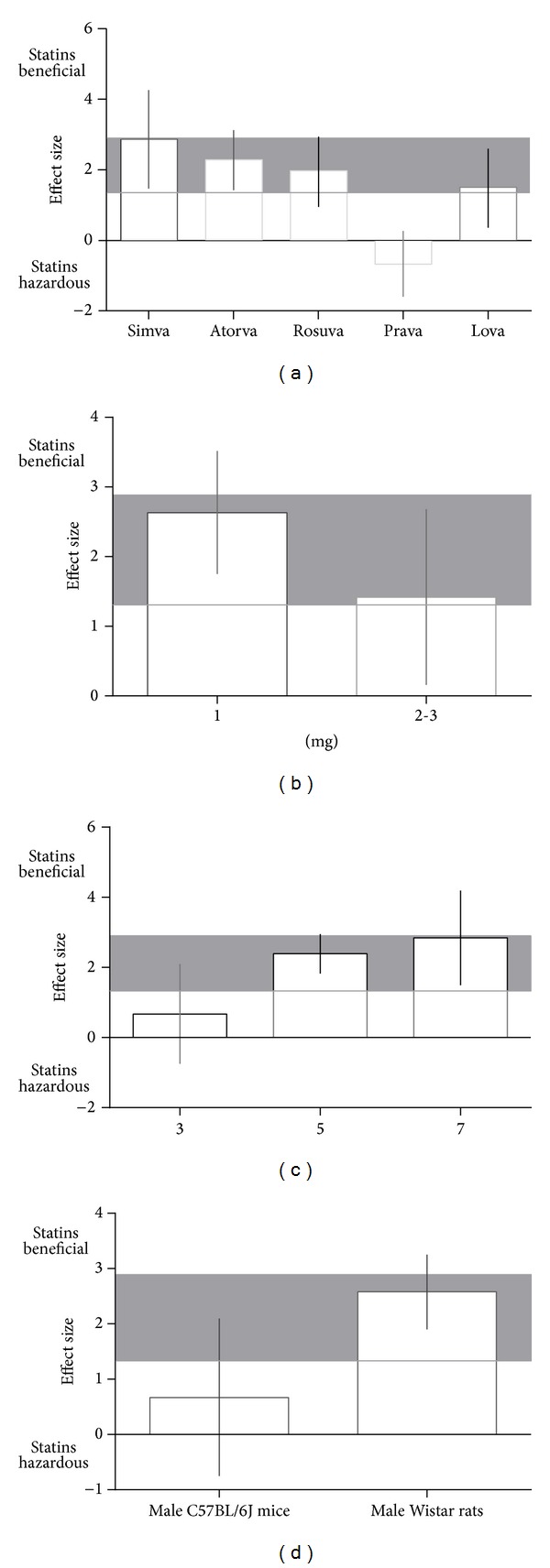
Effect size for retention memory stratified by (a) type of statin, (b) dose, (c) quality of study, and (d) animal species. Grey bands represent the 95% CI for the global estimated effect size.

Next, we sought to analyze the efficacy of different doses of statins on cognitive performance. For both acquisition and retention memory, significant beneficial effects were noted for all doses of statins, with a maximum effect at the lowest dose (−3.93, 95% CI: −6.74 to −1.12, Figure 3(b); 2.63, 95% CI: 1.75 to 3.52, Figure 4(b), resp.). However, no significant differences among doses were detected (*χ* ^*2*^ = 2.93, df = 1, and *P* = 0.09;  *χ* ^*2 *^= 2.37, df = 1, and *P* = 0.12, resp.).

The effect sizes for acquisition and retention memory were also examined relative to study quality score. No significant differences in effect sizes were observed between lower-scored and higher-scored studies (*χ* ^*2 *^= 10.63, df = 4, and *P* = 0.03;  *χ* ^*2*^ = 5.68, df = 2, and *P* = 0.06 for acquisition and retention memory, resp.). However, effect size for acquisition memory was maximum for studies with a quality score of 4 (−2.94, 95% CI: −4.04 to −1.85; Figure 3(c)), and effect size for retention memory was higher for studies with a quality score of 7 (2.84, 95% CI: 1.49 to 4.19; Figure 4(c)) than those with scores of 3 or 5.

For acquisition memory, effect size was similar for experiments using male Wistar rats and those using male C57BL/6J mice (*χ* ^*2 *^= 1.36, df = 1, and *P* = 0.24; Figure 3(d)). However, for retention memory, effect size was higher for studies using male Wistar rats (2.58, 95% CI: 1.90 to 3.52;  *χ* ^*2 *^= 5.6, df = 1, and *P* = 0.02; Figure 4(d)).

**Figure 5:**
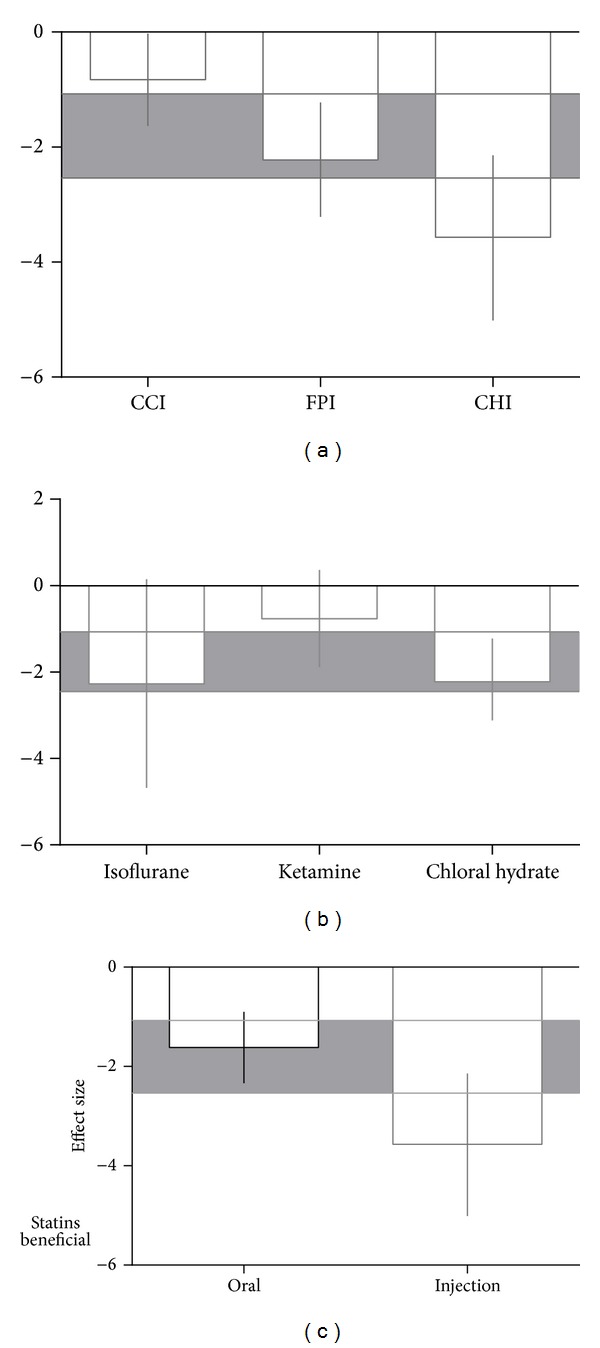
Effect size for acquisition memory stratified by (a) method of TBI induction, (b) anesthetic agent, and (c) route of drug delivery. Grey bands represent the 95% CI for the global estimated effect size.

**Figure 6:**
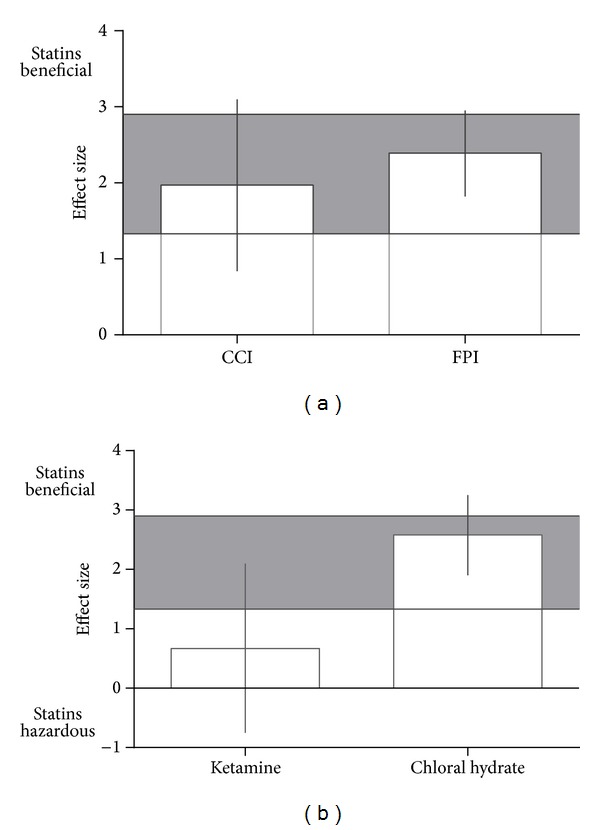
Effect size for retention memory stratified by (a) method of TBI induction and (b) anesthetic agent. Grey bands represent the 95% CI for the global estimated effect size.

Concerning anesthetic agents and types of TBI models, for acquisition memory, effect size was significantly higher in studies using closed head injury models (−3.57, 95% CI: −5.01 to −2.14;  *χ* ^*2 *^= 12.09, df = 2, and *P* = 0.002; Figure 5(a)) and those using isoflurane anaesthesia (−2.27, 95% CI: −4.68 to −0.15; *χ* ^*2 *^= 3.93, df = 2, and *P* = 0.14; Figure 5(b)). For retention memory, effect size was significantly higher in studies using fluid percussion injury models (2.39, 95% CI: 1.82 to 2.90; *χ* ^*2 *^= 0.42, df = 1, and *P* = 0.52; Figure 6(a)) and those using chloral hydrate anaesthesia (2.58, 95% CI: 1.90 to 3.25;  *χ* ^*2*^ = 5.60, df = 1, and *P* = 0.02; Figure 6(b)).

For acquisition memory, intraperitoneal administration (−3.57, 95% CI: −5.01 to −2.14; Figure 5(c)) was associated with a greater beneficial outcome than oral administration, but there were no significant differences between routes of administration (*χ* ^*2*^ = 5.69, df = 1, and *P* = 0.02).

### 3.6. Metaregression Analyses

Metaregression is an extension of subgroup analysis that allows investigation of the effect of continuous as well as categorical characteristics. In principle, metaregression also allows investigation of the effects of multiple factors simultaneously. The outcome variable is the effect estimate, and the explanatory variables are characteristics of studies that might influence effect size, which are often called “potential effect modifiers” or covariates.

To further explore heterogeneity among studies, metaregression was conducted for acquisition and retention memory. For retention memory, animal species, quality score, and type of anesthetic agent were significant sources of heterogeneity (*P* < 0.05). However, for acquisition memory, heterogeneity was independent of these factors (Table 5).

### 3.7. Publication Bias

**Figure 7:**
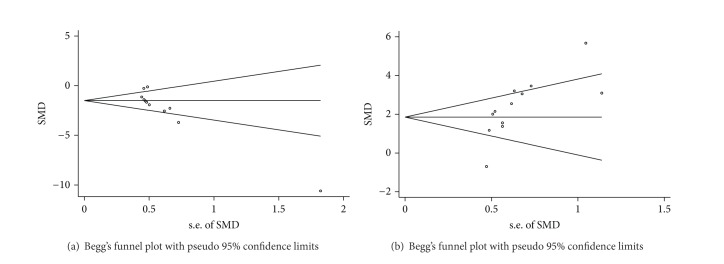
Funnel plot for acquisition memory (a) and retention memory (b).

Finally, we sought to identify the presence of small study effects, which may contribute to publication bias. Funnel plots show asymmetry for both acquisition and retention memory data, indicating evidence of small study effects (Figures 7(a) and 7(b); Egger regression, *P* < 0.0001 and *P* = 0.007, resp.).

### 3.8. Possible Drug Protection Mechanism Analysis

All studies selected during initial screening assessed the biological mechanisms of statin activity. Across studies, the neuroprotective effect of statins was attributed primarily to regulation of circulating endothelial progenitor cells and angiogenesis, increased neurogenesis and reduced neuronal degeneration, intravascular thrombosis and inflammation, and reduced microglial activation (Table 6).

## 4. Discussions

The results of animal experiments are used to inform decisions regarding the design and conduct of subsequent clinical trials. Systematic reviews of animal studies can allow such decisions to be based on the entirety of existing evidence that is synthesized in an unbiased manner. We therefore systematically reviewed and collated experimental evidence of the effect of statin administration before or after TBI in animal models, determined the efficacy of statin treatment in TBI, and explored the impact of study characteristics on statin efficacy.

Although there are some systematic reviews of pharmacological treatments (i.e., beta-2 receptor antagonists, progesterone) for TBI in animal models [44, 45], to our knowledge, this investigation is the first systematic review and meta-analysis of the efficacy of statins on cognitive deficits in animal models of TBI. Despite the presence of small effects and statistical heterogeneity among studies, our investigation shows that statins potentially exert neuroprotective effects in terms of improving cognitive outcome after TBI, with atorvastatin exerting the most protective effect on acquisition memory and simvastatin exerting the most protective effect on retention memory. Moreover, statin treatment provides better neuroprotection of acquisition memory for closed head injury. However, stratified analysis detected no significant influence of study quality, statin dose, animal species, drug delivery route, or anesthetic agent. Similar works [23, 46] have been performed in the context of experimental stroke, which demonstrate the neuroprotective effects of statins on animal stroke models in terms of reduced infarct volume and improved neurological severity score. Although stroke and TBI are different conditions, many aspects of their pathologies are similar, and these investigations provide further evidence of the neuroprotective effects of statins, thereby supporting their potential use for human TBI therapy.

We assessed the methodological quality of studies in accordance with previously described standards for preclinical development of neuroprotective drugs with minor modifications [22]. Overall, we found that the quality of the included studies was poor, as many failed to report blinded assessment of outcome or to determine a dose-response relationship, which are important issues that are generally required in clinical studies [47]. Moreover, lower quality studies showed a trend toward better acquisition memory outcomes. Therefore, the global estimated effect of statins on cognition may be overstated in low quality studies.

Furthermore, we found significant heterogeneity among study results. The main reasons for heterogeneity were the limited number of studies and the small sample sizes within those studies. Another important contribution to this heterogeneity may be the low quality of studies and potential bias of the studies selected for analysis [48]. To examine potential sources of heterogeneity, we performed metaregression analysis. Unfortunately, for retention memory, the adjusted *R* ^2^ was negative (data not shown) because the number of studies was small and the covariates explained less heterogeneity than would be expected by chance [49]. Therefore, it was not possible to accurately judge whether the heterogeneity we observed was independent of these factors, which made the analysis less reliable.

Our study has several limitations, which are also observed in previous systematic reviews of animal studies [44, 50, 51]. First, our analysis is only based on published data and did not take unpublished data into account; therefore publication bias should be considered. Second, we focused only on the effect of statins on cognitive deficits following TBI, largely due to insufficient data regarding histopathology such as lesion volume. Functional outcome, in combination with effects on histopathology, may be as important in terms of assessing benefit of potential neuroprotective drugs [52]. Third, the current findings may be influenced by the selective inclusion of studies that examined only male rodents. Although intact male and female animals should be examined prior to clinical investigation, the small number of studies that examined intact females, combined with potential sex differences in outcome [53], meant that their inclusion could have muddied the results [51]. Fourth, a variety of different metrics were used (e.g., pressure, weight, and velocity) to evaluate TBI severity, and no studies specified the degree of severity (e.g., mild, moderate, or severe). Thus, the results of different studies could be more accurately compared if injury severity is reported in a consistent manner. Fifth, although we found that statin treatment can have beneficial effects in animal models of TBI, the majority of studies used only controlled cortical impact or fluid percussion injury models. However, any one animal model may not fully recapitulate all the aspects of secondary injury development observed in humans with TBI [54], thereby limiting the extent to which this experimental research translates to a clinical population. Finally, there were large numbers of studies that failed to report, or provide upon written request, the necessary data, which therefore had to be derived from graphs. Although we enlarged the graphs, and data were independently extracted by two investigators, this technique can be imprecise. Moreover, extracting multiple pieces of information from a single publication has the potential to introduce bias into systematic reviews because the results are generated by the same investigators.

To improve the transition from animal experiments to human clinical trials, future animal studies of statins or other drugs should improve their methodological reporting and quality control as follows: (1) additional appropriate and standardized TBI models are needed to evaluate the impact of promising pharmacological interventions; (2) treatment efficacy should be tested in both sexes and different species (i.e., rabbits, cats, or gyrencephalic primates); (3) researchers should consult and follow the ARRIVE guidelines [55, 56] when designing studies and report full methodological details to allow others to reproduce and validate their results and to enable more accurate reviews and meta-analyses; (4) other short- and long-term outcomes such as lesion volume, brain edema, blood-brain barrier permeability, and depression-like behavior should also be examined.

## 5. Conclusions

Despite its limitations, this systematic review and meta-analysis demonstrates that statins could reduce cognitive deficits in animal models of TBI. A fundamental assumption is that the results of animal studies, if performed well enough, will predict effects in humans. However, promising neuroprotective drugs previously identified as effective in animal TBI models have failed in Phase II or III clinical trials [54]. Therefore, without rigorous, robust, and detailed preclinical evaluation, it is unlikely that novel neuroprotective drugs will prove effective when tested in large, time-consuming, and expensive human clinical trials, thereby warranting further well-designed and well-reported experimental animal studies.

## Figures and Tables

**Table 1:** Key search terms used in database searches.

Traumatic brain injury	Statins	
Traumatic brain injury	Statins	Compactin
Traumatic brain injuries	Statin	Mevinolin
TBI	Atorvastatin	Rosuvastatin
Head injury	Dalvastatin	Simvastatin
Head injuries	Fluvastatin	Pitavastatin
Brain injury	Lovastatin	Pravastatin
Brain injuries	Mevastatin	
Injury brain	HMG-CoA reductase inhibitors
Injuries brain	Hydroxymethylglutaryl-CoA reductase inhibitors
Head trauma	Hydroxymethylglutaryl-coenzyme A inhibitors
	Hydroxymethylglutaryl-CoA inhibitors

**Table 2:** Criteria for study inclusion/exclusion.

Inclusion criteria	Exclusion criteria
(1) Statins were administered.	(1) Statins were not administered.
(2) Experimental TBI was induced in rodents.	(2) No control group was used.
(3) Cognitive function was measured by the MWM.	(3) Nonimpact (e.g., cortical ablation) or penetrating (e.g., missile-induced) TBI was performed.
(4) Male rodents (i.e., rats or mice) were used.	(4) Treatment group was administered another neuroprotective agent in addition to a statin.
(5) Article was published in English or Chinese language.	(5) Other types of animals (e.g., sheep, cats, and dogs) were used.
(6) A TBI treatment group was treated with a pharmacological agent, and a control group was administered a placebo after injury.	(6) Only biochemical or physiological outcomes of treatment efficacy were assessed.
	(7) Samples included female rodents.
	(8) Duplicate publications.

**Table 3:** The CAMARADES quality items.

Author	(1)	(2)	(3)	(4)	(5)	(6)	(7)	(8)	(9)	(10)	Quality score
Abrahamson et al., 2009 [24]	*√*	*√*			*√*		*√*			*√*	5
Chauhan and Gatto, 2011 [16]	*√*						*√*			*√*	3
Indraswari et al., 2012 [25]	*√*	*√*	*√*	*√*	*√*		*√*		*√*	*√*	8
Lu et al., 2004 [26]	*√*	*√*		*√*	*√*		*√*	*√*		*√*	7
Lu et al., 2007 [13]	*√*	*√*		*√*	*√*		*√*	*√*		*√*	7
Wang et al., 2012 [27]	*√*	*√*					*√*	*√*		*√*	5
Wang et al., 2007 [28]	*√*	*√*			*√*					*√*	4
Wu et al., 2008 [10]	*√*	*√*		*√*	*√*		*√*	*√*		*√*	7
Jin et al., 2013 [29]	*√*	*√*						*√*		*√*	4
Liu, 2009 [30]	*√*	*√*					*√*	*√*		*√*	5
Zhang et al., 2012 [31]	*√*	*√*		*√*				*√*		*√*	5

Note: (1) peer reviewed publication; (2) presence of randomization of subjects into treatment groups; (3) assessment of dose-response relationship; (4) blinded assessment of behavioural outcome; (5) monitoring of physiological parameters such as body temperature; (6) calculation of necessary sample size to achieve sufficient power; (7) statement of compliance with animal welfare regulations; (8) avoidance of anaesthetic agents with marked intrinsic neuroprotective properties (e.g., ketamine); (9) statement of potential conflict of interests; (10) use of a suitable animal model.

**Table 4:** Characteristics of included studies.

Study	Animal species	Injury model	Main experimental groups	Method/dose of statin administration	Anesthetic agent	Time of statin administration	Time of outcome measurement	Quality score
Abrahamson et al., 2009 [24]	Male APP^NLh/NLh^ mice	CCI	(1) TBI + vehicle (*n* = 5-6) (2) TBI + simvastatin (*n* = 5-6)	Orally, 3 mg/kg/d	Isoflurane	3 h after injury	10–14 d after injury	5

Chauhan and Gatto, 2011 [16]	Male C57BL/6J mice	CCI	(1) TBI+ saline (*n* = 8) (2) TBI + pravastatin (*n* = 12) (3) TBI + lovastatin (*n* = 9) (4) TBI + simvastatin (*n* = 8)	Orally, 2 mg/kg/d for 8 weeks	Ketamine	immediately after injury	7-8 weeks after injury	3

Indraswari et al., 2012 [25]	Male C57BL/6J mice	CCI	(1) TBI + saline (*n* = 12) (2) TBI + rosuvastatin (*n* = 12)	Orally, 1 mg/kg/d for 5 d	Isoflurane	within 1 h after injury	31–34 d after injury	8

Lu et al., 2004 [26]	Male Wistar rats	CCI	(1) TBI + saline (*n* = 4) (2) TBI + atorvastatin (*n* = 4)	Orally, 1 mg/kg/d for 7 d	Chloral hydrate	1 d after injury	1, 4, 8, and 15 d after injury	7

Lu et al., 2007 [13]	Male Wistar rats	CCI	(1) TBI + saline (*n* = 10) (2) TBI + atorvastatin (*n* = 10) and (1) TBI + saline (*n* = 10) (2) TBI + atorvastatin (*n* = 10) (3) TBI + simvastatin (*n* = 10)	orally 1 mg/kg/d for 14 d	Chloral hydrate	1 d after injury	11–15 and 31–35 d after injury	7

Wang et al., 2012 [27]	Male Wistar rats	FPI	(1) TBI + control (*n* = 10) (2) TBI + atorvastatin (*n* = 10)	Orally, 1 mg/kg/d for 14 d	Chloral hydrate	1 h after injury	21–25 d after injury	5

Wang et al., 2007 [28]	Male C57Bl/6J mice	CHI	(1) TBI + vehicle (*n* = ?) (2) TBI + simvastatin (*n* = ?) (3) TBI + atorvastatin (*n* = ?)	Subcutaneous injection, 20 mg/kg/d for 17 d	Isoflurane	Unclear	21–25 d after injury	4

Wu et al., 2008 [10]	Male Wistar rats	CCI	(1) TBI + saline (*n* = 8) (2) TBI + simvastatin (*n* = 8)	Orally, 1 mg/kg/d for 14 d	Chloral hydrate	1 d after injury	31–35 d after injury	7

Jin et al., 2013 [29]	Male Wistar rats	FPI	(1) TBI + saline (*n* = 12) (2) TBI + atorvastatin (*n* = 12)	Orally, 1 mg/kg/d for 14 d	Chloral hydrate	Within 1 h after injury	21–25 d after injury	4

Liu, 2009 [30]	Male Wistar rats	FPI	(1) TBI + saline (*n* = 11) (2) TBI + simvastatin (*n* = 13)	Orally, 20 mg/kg/d, unclear treatment duration	Chloral hydrate	1 d after injury	21–25 d after injury	5

Zhang et al., 2012 [31]	Male Wistar rats	FPI	(1) TBI + saline (*n* = 12) (2) TBI + atorvastatin (*n* = 12) (3) TBI + simvastatin (*n* = 12) (4) TBI + rosuvastatin (*n* = 12)	Orally, atorvastatin/simvastatin: 2 mg/kg/d for 21 d, rosuvastatin: 1 mg/kg/d for 21 d	Chloral hydrate	Within 1 d after injury	22–28 d after injury	5

Note: CCI: controlled cortical impact; FPI: fluid percussion injury; CHI: closed head injury.

**Table:** (a) Acquisition memory

Covariates	Coef.	Std. err.	*t*	*P* > |*t*|	[95% conf. interval]	
Species	−1.218926	1.425615	−0.86	0.415	−4.443892	2.00604
Quality	−.1059611	.5134567	−0.21	0.841	−1.267481	1.055559
Route	1.794537	2.46653	0.73	0.485	−3.785142	7.374215
Statins						
Atorva	−4.098033	2.606752	−1.57	0.167	−10.47653	2.28046
Lova	.1509175	3.110633	0.05	0.963	−7.460527	7.762362
Prava	−1.058402	2.688579	−0.39	0.707	−7.637118	5.520313
Simva	−1.752053	2.462111	−0.71	0.503	−7.776621	4.272516
Dose						
1 mg	−2.813334	2.734279	−1.03	0.338	−9.278877	3.652209
2 mg	−.0024291	2.536972	−0.00	0.999	−6.001415	5.996557
20 mg	−1.646112	2.854527	−0.58	0.582	−8.395994	5.103771
Anaesthetic used						
Chloral hydrate	−.3426693	2.006333	−0.17	0.869	−4.969281	4.283942
Ketamine	1.502998	2.222102	0.68	0.518	−3.621178	6.627174
Injury model						
CCI	2.776583	2.521451	1.10	0.303	−3.037893	8.591059
FPI	1.121622	2.456371	0.46	0.660	−4.54278	6.786024

**Table:** (b) Retention memory

Covariates	Coef.	Std. err.	*t*	*P* > |*t*|	[95% conf. interval]	
Species	−2.045101	.7652448	−2.67	0.023	−3.750172	−.3400288
Dose	−1.358647	.8065987	−1.68	0.123	−3.155861	.4385665
Quality	.5416233	.2248974	2.41	0.037	.0405206	1.042726
Anaesthetic used	2.045101	.7652448	2.67	0.023	.3400288	3.750172
Injury model	−.5074523	.9452993	−0.54	0.603	−2.61371	1.598806
Statins						
Atorva	.4747928	1.355065	0.35	0.736	−2.729427	3.679012
Lova	−.4478023	1.743837	−0.26	0.805	−4.571322	3.675718
Prava	−2.709569	1.71473	−1.58	0.158	−6.764262	1.345123
Simva	.9555847	1.387517	0.69	0.513	−2.325371	4.23654

**Table 6:** Possible protective mechanisms of statins.

Possible protective mechanisms of statins	Studies
Blunted TBI-induced increases in amyloid beta protein, reduced hippocampal tissue damage, and microglial activation	[24]
Restored axonal integrity	[16]
Downregulation of inflammatory gene expression, reduced neuronal degeneration, preserved neuronal density, and reduced microgliosis	[25]
Reduction of intravascular thrombosis, increased cerebral microvascular patency and integrity	[26]
Increased neurogenesis in the dentate gyrus, reduced delayed neuronal death in the hippocampal CA3 region	[10, 13]
Reduced hippocampal degeneration, improved cerebral blood flow	[28]
Regulation of circulating endothelial progenitor cells and angiogenesis	[27, 29–31]
